# Delta-9-Tetrahydrocannabinol Blocks Bone Marrow-Derived Macrophage Differentiation through Elimination of Reactive Oxygen Species

**DOI:** 10.3390/antiox13080887

**Published:** 2024-07-23

**Authors:** Taylor H. Carter, Chloe E. Weyer-Nichols, Jeffrey I. Garcia-Sanchez, Kiesha Wilson, Prakash Nagarkatti, Mitzi Nagarkatti

**Affiliations:** Department of Pathology Microbiology and Immunology, University of South Carolina School of Medicine, Columbia, SC 29209, USA; carterth@uscmed.sc.edu (T.H.C.); cweyer@uscmed.sc.edu (C.E.W.-N.); garciasj@uscmed.sc.edu (J.I.G.-S.); kiesha.wilson@uscmed.sc.edu (K.W.); prakash@mailbox.sc.edu (P.N.)

**Keywords:** differentiation, tetrahydrocannabinol, M-CSF, ROS

## Abstract

Macrophages are vital components of the immune system and serve as the first line of defense against pathogens. Macrophage colony-stimulating factor (M-CSF) induces macrophage differentiation from bone marrow-derived cells (BMDCs). Δ9-tetrahydrocannabiol (THC), a phytocannabinoid from the *Cannabis* plant, has profound anti-inflammatory properties with significant effects on myeloid cells. To investigate the effect of THC on macrophage differentiation, we cultured BMDCs with M-CSF in the presence of THC. Interestingly, THC markedly blocked the differentiation of BMDCs into CD45 + CD11b + F4/80+ macrophages. The effect of THC was independent of cannabinoid receptors CB1, and CB2, as well as other potential receptors such as GPR18, GPR55, and Adenosine 2A Receptor. RNA-seq analysis revealed that the THC-treated BMDCs displayed a significant increase in the expression of NRF2-ARE-related genes. KEGG pathway analysis revealed that the expression profiles of THC-treated cells correlated with ferroptosis and glutathione metabolism pathways. Fluorescence-based labile iron assays showed that the THC-treated BMDCs had significantly increased iron levels. Finally, THC-exposed BMDCs showed decreased levels of intracellular ROS. THC has the unique molecular property to block the Fenton Reaction, thus preventing the increase in intracellular ROS that is normally induced by high iron levels. Together, these studies demonstrated that THC blocks M-CSF-induced macrophage differentiation by inhibiting ROS production through both the induction of NRF2-ARE-related gene expression and the prevention of ROS formation via the Fenton Reaction.

## 1. Introduction

Myeloid cells play a vital role in metabolism as well as immune and physiological development [1]. Various growth factors support myeloid cell differentiation from hematopoietic stem cells (HSCs). Macrophage colony-stimulating factor (M-CSF) is one such growth factor that drives the differentiation and proliferation of macrophages from HSCs without altering their activation status [2]. M-CSF dysregulation has been implicated in the pathogenesis of multiple disease states, including arthritis, atherosclerosis, and renal inflammation [3,4,5,6]. M-CSF binds to CSFR1, a G-protein-coupled receptor (GPCR), which results in the activation of PU.1 transcription factor and subsequently induces macrophage differentiation [7,8]. The M-CSF/CSFR1 axis is currently the focal point for drug development targeting M-CSF differentiation in disease states [2,3,4,5,6,7,8,9]. Outside of transcriptional regulation of this system, studies have shown that reactive oxygen species (ROS) are also required for M-CSF-induced macrophage differentiation [10,11]. The role of iron in the redox state of macrophages is currently a growing field of research which aims to better understand iron-based cell death, known as ferroptosis [12,13,14].

The reliance of macrophage differentiation upon the M-CSF/CSFR1 axis and iron-dependent intracellular ROS production sets the stage for Δ9-Tetrahydrocannabinol (THC) as a potential target molecule for the interruption of this physiological process. THC, a phytochemical from the *Cannabis* plant, has potent immunosuppressive and anti-inflammatory effects [15,16,17]. THC’s ability to dampen macrophage response has been well documented in various disease models [15,16,18]. Historically, THC was thought to operate through GPCRs, namely Cannabinoid Receptor 1 and 2 (CB1 and CB2, respectively). However, recent evidence has demonstrated that THC’s immuno-modulatory effects persist despite the complete knock-out or inhibition of both CB1 and CB2 [19,20]. With the expansion of THC-focused research over the last decade, the field has established the involvement of other GPCRs, such as GPR18, GPR55, A2aR, and TRPV2, in THC-mediated signaling pathways [21,22,23,24]. Not only does THC directly interact with these GPCRs, but these receptors can also form heterodimers or homodimers, which can result in a crosstalk between the receptors [25,26,27]. In addition to these ligand–receptor interactions, THC can also act through receptor-independent mechanisms. One such example includes how THC exhibits potent antioxidant effects through electron abstraction at the phenol position [28,29].

Given THC’s strong immunomodulatory properties, this compound was selected as the focus of this study to investigate how it affects HSCs in the early stages of myeloid differentiation. M-CSF-induced macrophage differentiation from bone marrow-derived cells (BMDCs) is classically used as an in vitro model for macrophage studies. LPS is normally added after initial macrophage differentiation to further activate and polarize the macrophages [30,31].

In this present study, we investigated THC’s ability to block macrophage differentiation, the potential role of THC’s receptor-independent mechanisms, and the function of ROS and iron in macrophage differentiation. Intriguingly, we found that THC blocks macrophage differentiation via a receptor-independent mechanism by eliminating ROS through both the activation of the NRF2-ARE system and the functional inhibition of iron involved in the ROS-producing Fenton Reaction.

## 2. Methods

### 2.1. Mice

Eight-week-old female wild-type C57BL/6 mice were purchased from Jackson Laboratories (Bar Harbor, ME, USA). CB1KO constitutive knockout mice were gifted from Dr. James Pickel (NIH National Institute of Mental Health Transgenic Core Facility, Bethesda, MD, USA). CB2KO constitutive knockout mice (B6.129P2-Cnr2tm1Dgen/J) were obtained from Jackson Laboratories (JAX 005786). CB1/CB2 KO mice were bred in-house, and colonies were maintained at the University of South Carolina School of Medicine animal facility. The University of South Carolina Institutional Animal Care and Use Committee (IACUC) approved all experiments. All animals were housed within the University Of South Carolina School Of Medicine’s AAALAC-accredited animal facility under temperature-controlled and specific pathogen-free conditions (Columbia, SC, USA). Mice were provided ad libitum access to water and normal chow. Mice were euthanized via isoflurane overdose and cervical dislocation was used as a secondary euthanasia measure.

### 2.2. Reagents

THC was obtained from Cayman Chemical (Ann Arbor, MI, USA); GPCR inhibitors ML193, PSB CB5, SET 2, SCH442416, and Deferoxamine from Tocris (Minneaoikus, MN, USA); Pertussis Toxin (PTX) from List Biological Laboratories (Campbell, CA, USA); Recombinant Murine M-CSF and Annexin V Apoptosis Detection Kit from Biolegend (San Diego, CA, USA); PhenGreen SK (PGSK) and CellROX Deep Red and Sytox Blue from Thermo Fisher (Waltham, MA, USA); Penicillin/Streptomycin, FBS, phosphate-buffered saline (PBS) from VWR (West Chester, PA, USA).

### 2.3. Cell Preparation

Tibia and Femur bone marrow was immediately collected after euthanasia of mice and suspended in DMEM/F12 media from Corning (Corning, NY, USA), which was supplemented with 10% FBS and 1% Pen/Strep. Bone marrow was mechanically homogenized, filtered through 70 µM filters and counted using Bio-Rad’s TC20 Automated Cell Counter (Hercules, CA, USA). Cells (1 × 10^6^) per well were added to a 24-well Corning culture plate. THC dissolved in ethanol suspension or vehicle (ethanol) was added to each well followed by M-CSF to achieve a final concentration of 1 µg/mL M-CSF/well. In experiments utilizing receptor antagonists, they were added directly before THC addition. THC was applied at a concentration of 3, 15, or 30 µM. Respective concentrations are listed in figure legends. Plates were incubated at 37 °C with 5% CO_2_. Cells were collected at 48 h for RNA-seq, viability staining, and ROS assay. At 96 h, Lipopolysaccharide (LPS) serotype 026-B6 was added at a concentration of 100 ng/mL (Invitrogen, Waltham, MA, USA). At 120 h, cells were collected for flow cytometry.

### 2.4. Flow Cytometry

Cells were washed in FACS buffer and Fc-blocked (TruStain FcX anti-mouse CD16/32; clone 93) for 10 min. Cells were tagged with fluorescently labeled monoclonal antibodies (mAbs) purchased from BioLegend (APC/Cy7 labeled anti-CD45, AF700 labeled anti-CD68, BV650 labeled anti-Ly6C, FITC labeled anti-Ly6G), BD Biosciences (San Jose, CA, USA) (BUV661 labeled anti-CD11b), and Bio-Rad (StarBright Blue 615 labeled anti-F4/80). Stained cells were washed with FACS buffer twice then analyzed on a BD FACSymphony A5SE flow cytometer. The FlowJo analysis software package v10.8.1 from BD Biosciences was used to analyze FCS files (Ashland, OR, USA).

### 2.5. RNA-Seq

For RNA-seq, the bone marrow cells from groups of 3 mice was plated with M-CSF with either THC or vehicle. At 48 h, cells were collected from wells and put in Qiazol from Qiagen (Valencia, CA, USA). Total RNA was purified using Qiagen RNeasy kit. RNA library was prepared using NEBNext Ultra II RNA Library Prep Kit then NEBNext Multiplex Oligos for Illumina, according to manufacturer’s protocol (Ipswich, MA, USA). Samples were sequenced on Illumina (San Diego, CA, USA) NextSeq 550 instrument. FastQ files were input into the Galaxy software application v24.1.0 [32]. The sequences were first trimmed with Cutadapt. The sequences were then aligned and input into DESeq2 analysis. The DESeq2 output was used to create the volcano plot, and the resulting VST-normalized counts were used to create a heat map with Heatmap2 v3.1.3.1.

### 2.6. Fluorescent Assays

For apoptosis detection at 48 h, cells were stained with BioLegend’s FITC Annexin V Apoptosis Detection Kit with PI. Live cells were determined by staining with BioLegend’s Zombie UV. Stained cells were run on BD FACSCelesta flow cytometer and populations gated in the FlowJo software v.10.8.1 package. For iron assays, PGSK was directly added to wells at either 24 or 48 h. After a 30 min incubation period, cells were run on BD FACSymphony A5SE, and mean fluorescent intensity was calculated in FlowJo. For the ROS assay, CellROX Deep Red solution was added directly to wells at 24 and 48 h. Cells were washed three times in 1× PBS and run on the BD FACSymphony A5SE. FlowJo software was used to gate Sytox- populations (live cells) and mean fluorescent intensity was calculated.

### 2.7. Statistical Analyses

All data were analyzed using the GraphPad Prism Version 9.0 software package (San Diego, CA, USA). Unless otherwise noted in figure legends, analyses were performed using one-way analysis of variance (ANOVA) tests and are presented as mean ± SEM. Statistical tests and number of replicates (*n*) are noted in the figure legends. Levels of statistical significance were assigned according to the following cutoffs: * *p* < 0.05, ** *p* < 0.01, *** *p* < 0.001, and **** *p* < 0.0001.

## 3. Results

### 3.1. THC Blocks Macrophage Differentiation in a Dose-Dependent Manner

**Figure 1:**
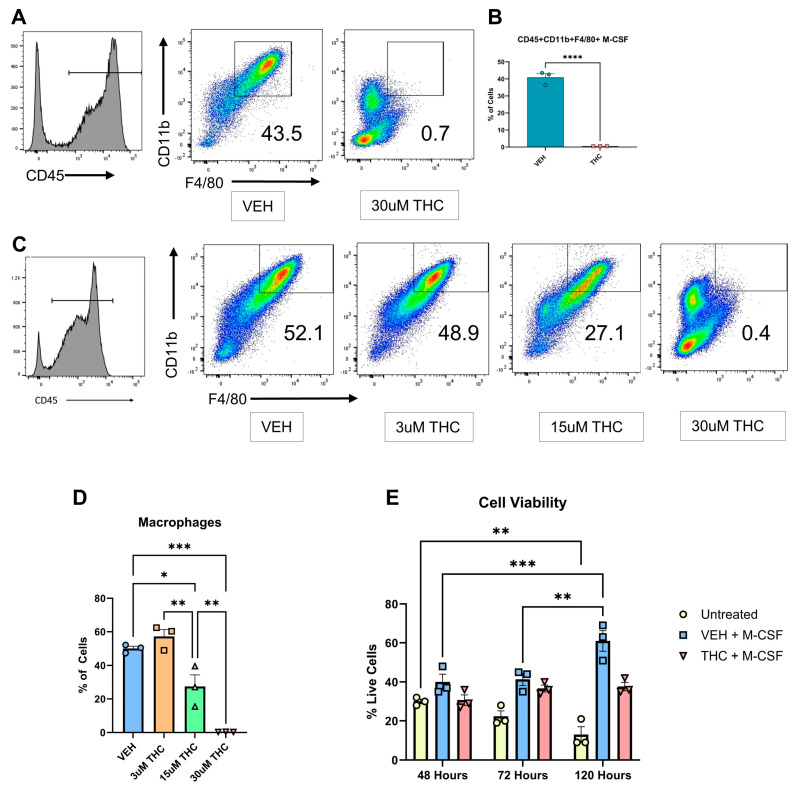
In vitro differentiation of BMDCs into macrophages. (**A**) Representative flow cytometry pseudocolor plots for BMDCs following culture with M-CSF and the addition of LPS at 96 h. CD45+ gated cells were further analyzed for the expression of CD11b + F4/80+ cells, the percentage of which is shown in each histogram. Unpaired *t*-test was performed between the two groups. (**B**) Graphical representation of the percentages of macrophages in vehicle and THC-treated groups at 120 h by flow cytometry. (**C**) Representative flow cytometry pseudocolor plot for CD45 + CD11b + F4/80+ macrophages cultured with different concentrations of THC. (**D**) Graphical representation of the percentages of macrophages in THC dose-dependent response assay. (**E**) Cell viability as measured by cell counter at 48, 72, and 120 h. Two-way ANOVA was performed. (**A**–**E**) n = 3, levels of statistical significance were assigned according to the following cutoffs: * *p* < 0.05, ** *p* < 0.01, *** *p* < 0.001, and **** *p* < 0.0001.

Although THC’s effect on mature macrophage functions has been well documented, its effect on macrophage differentiation is not well defined [15,16,18]. To investigate any potential THC-mediated effects on macrophage differentiation, THC was added to bone marrow-derived cells (BMDCs) along with M-CSF, and the differentiation status was examined over a 120 h period. Additionally, 100 ng/mL Lipopolysaccharide (LPS) was added at 96 h to induce macrophage activation and polarization [30]. Flow cytometric analysis revealed that THC inhibited macrophage differentiation as seen by a significantly lower percentage of CD45 + CD11b + F4/80+ cells (Figure 1A,B). The gating strategy used for flow cytometric analysis has been shown in Figure S1. To confirm THC’s effect on macrophage differentiation, a dose-dependent response assay was run utilizing 3 µM, 15 µM, and 30 µM THC under the same M-CSF and LPS culture conditions (Figure 1C,D). THC caused a dose-dependent decrease in the differentiation of BMDCs into CD45 + CD11b + F4/80+ macrophages (Figure 1C,D). To rule out the possibility that THC was causing changes in cell viability, we measured the cell viability at different time points and found that while untreated cells showed a decrease in viability at 120 h when compared to 48 h, cells cultured with M-CSF showed an increase in cell viability, while cells cultured with M-CSF + THC showed similar cell viability at 120 h when compared to 48 h of culture (Figure 1E). It has previously been shown that 30 µM THC alters the susceptibility of M-CSF-induced macrophage to HIV-1 infection function [33]. Given this evidence and the results of our dose-dependent experiments, a concentration of 30 µM THC was used throughout the remainder of this study.

### 3.2. Identifying the Receptors through Which THC Suppresses Macrophage Differentiation

**Figure 2:**
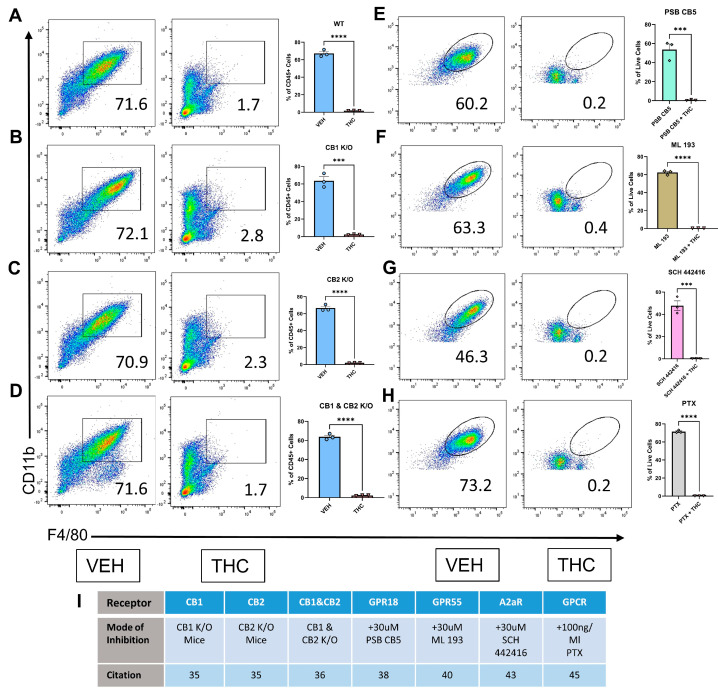
Identifying potential receptors through which THC blocks macrophage differentiation. (**A**–**H**) Representative flow and graphical representation of macrophage populations at 120 h between VEH- and THC-treated groups. Y-axis on flow plot is CD11b; X-axis is F4/80. (**A**–**D**) Preliminary flow gating shown in Figure S1A. (**E**–**H**) Preliminary flow gating shown in Figure S1B. (**A**) Wild-type mice; (**B**) CB1 knockout mice; (**C**) CB2 knockout mice; (**D**) CB1 and CB2 knockout mice; (**E**) GPR18 antagonist PSB CB5; (**F**) GPR55 antagonist ML 193; (**G**) A2aR antagonist SCH 442416; (**H**) G-PCR inhibitor PTX; (**I**) List of receptor inhibition methods and related citation. (**A**–**H**) n = 3, unpaired *t*-test was performed, levels of statistical significance were assigned according to the following cutoffs: *** *p* < 0.001, and **** *p* < 0.0001.

Δ9-THC is primarily regarded as a CB1/CB2 partial agonist [34]. To establish the role of CB1 and CB2 in THC’s ability to attenuate macrophage differentiation, constitutive CB1KO, CB2KO, and CB1/CB2 double KO mice were used [35,36]. We used the same culture conditions as used for BMDCs from wild-type C57BL/6 mice (30 µM THC and 1 µg/mL M-CSF at 0 h, 100 ng/mL LPS at 96 h), and the flow cytometry was run at 120 h. THC was effective in blocking macrophage differentiation, but surprisingly, THC was also highly effective in blocking macrophage differentiation in CB1KO, CB2KO, and CB1/CB2 double-KO mice (Figure 2A–D). Preliminary flow gating strategies shown in Figure S1A. These results suggested that the ability of THC to block macrophage differentiation was independent of CB1 and CB2 receptors.

It has also been shown that THC can mediate its effects through GPR18 in a CB1-independent manner [37]. Given this, we selected PSB CB5, a GPR18 antagonist [38], to investigate if this would block the effect of THC on macrophage differentiation. The addition of PSB CB5 to the BMDC cultures did not affect THC’s ability to block macrophage differentiation (Figure 2E). GPR55 is regarded as a novel cannabinoid receptor with a wide range of ligands and a response profile that differs from classical CB1 and CB2 signaling [21]. ML-193 is a GPR55 antagonist that has previously been used to block cannabinoid-mediated GPR55 signaling [39]. The addition of ML-193 did not affect THC’s ability to block macrophage differentiation (Figure 2F). We then looked at the potential role of Adenosine 2A Receptor, or A2AR in this phenomenon. A2AR and the endocannabinoid system have a close relationship, with A2AR often forming heterodimers with CB1 [40]. Some of THC’s behavioral effects have been shown to be altered in A2AR KO mice [41]. The addition of SCH442416 [42], an A2AR antagonist, alongside THC did not affect THC’s ability to block macrophage differentiation (Figure 2G). Finally, to confirm that any unknown or minor THC-binding GPCRs were not responsible for THC’s differentiation-blocking effects, Pertussis Toxin (PTX) was applied to disrupt GPCR protein coupling and, therefore, all GPCR ligand-mediated response [10,43]. The addition of PTX alongside THC had no effect on THC’s ability to disrupt macrophage differentiation (Figure 2H). The gating strategy for Figure 2E–H is shown in Figure S1B. Panel I shows the approach used for studying various potential receptors, doses of agents used, and citations on which the doses were selected.

### 3.3. RNA-Seq Analysis of BMDCs

**Figure 3:**
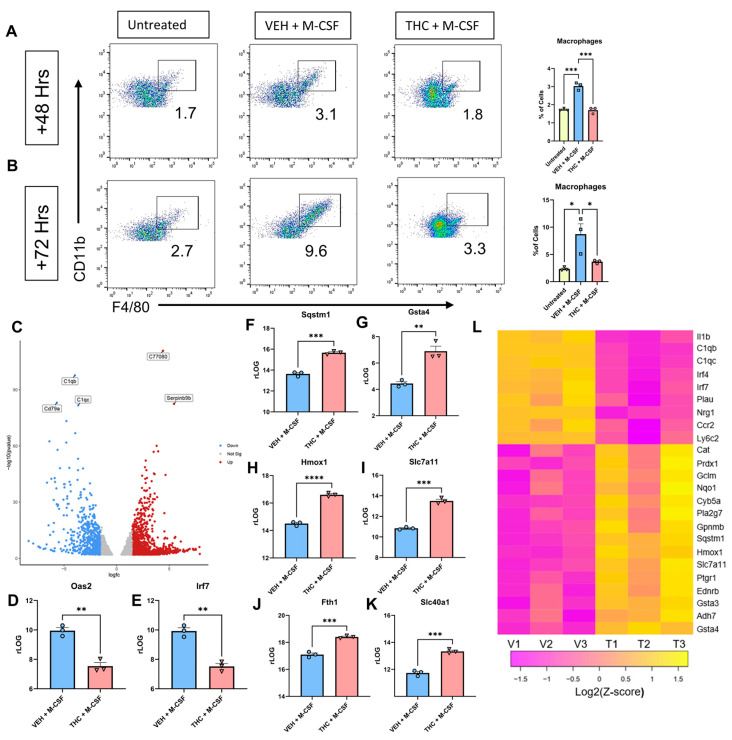
Analysis of BMDCs 48 h after culture with M-CSF. (**A**) Representative flow cytometry pseudocolor plot and graphical representation of CD45 + CD11b + F4/80+ macrophages at 48 h. Preliminary gating shown in Figure S1C. (**B**) Representative flow cytometry pseudocolor plot and graphical representation for CD45 + CD11b + F4/80+ macrophages at 72 h. Preliminary gating shown in Figure S1D. (**C**) Volcano plot of DEGS between VEH + M-CSF- and THC + M-CSF-treated groups at 48 h. (**D**,**E**) Graphical representation of rLOG normalization counts for VEH + M-CSF- and THC + M-CSF-treated groups at 48 h. (**F**–**K**) Graphical representation of rLOG normalization counts for ARE and iron-related genes between VEH + M-CSF- and THC + M-CSF-treated groups at 48 h. (**L**) Heat map of DEGs between VEH + M-CSF- and THC + M-CSF-treated groups at 48 h. (**A**,**B**,**D**–**I**) n = 3, levels of statistical significance were assigned according to the following cutoffs: * *p* < 0.05, ** *p* < 0.01, *** *p* <0.001, and **** *p* < 0.0001. (**D**–**I**) Unpaired *t*-test was performed between the two groups.

Next, we were interested in studying the transcriptional regulation of macrophage differentiation in the presence of THC at the early time points of their differentiation. To that end, we analyzed Veh + M-CSF- or THC + M-CSF-treated cultures for macrophage differentiation at 48 and 72 h (Figure 3A,B). The gating strategy is shown in Figures S1C and S1D, respectively. At 48 h, the cells cultured with Veh + M-CSF showed early signs of macrophage differentiation while THC + M-CSF cultures showed decreased macrophage differentiation. Thus, we investigated the transcriptional profiles of these cells at 48 h of culture. To achieve this, BMDCs were collected from three separate mice, and each sample was individually plated with either 30 µM THC or vehicle and 1 µM/mL M-CSF or untreated. At 48 h, the cells were collected and RNA-seq was performed. A volcano plot created off *p*-value (<0.05) and Log2FC (>1.5) displayed 1000s of differentially expressed genes (DEGs) (Figure 3C). A heat map was created looking at influential genes, taking read count into consideration and then selecting for Log2FC. The VEH + M-CSF group saw a significant increase in genes that have been shown to be upregulated during M-CSF-induced macrophage differentiation [30], such as *Irf4*, *Irf7*, *Plau*, and *Nrg1* (Figure 3L) while THC + M-CSF-treated cells showed a decreased expression of these genes. Irf4 has also been shown to influence M-CSF-driven macrophage polarization [44]. The VEH + M-CSF group also had an increase in genes seen in monocytes such as *Ccr2* and *Ly6c2* [45], while THC reversed the expression levels (Figure 3L). In all, the VEH + M-CSF group displayed an expression profile expected for M-CSF-induced macrophage differentiation from murine bone marrow [30].

**Figure 4:**
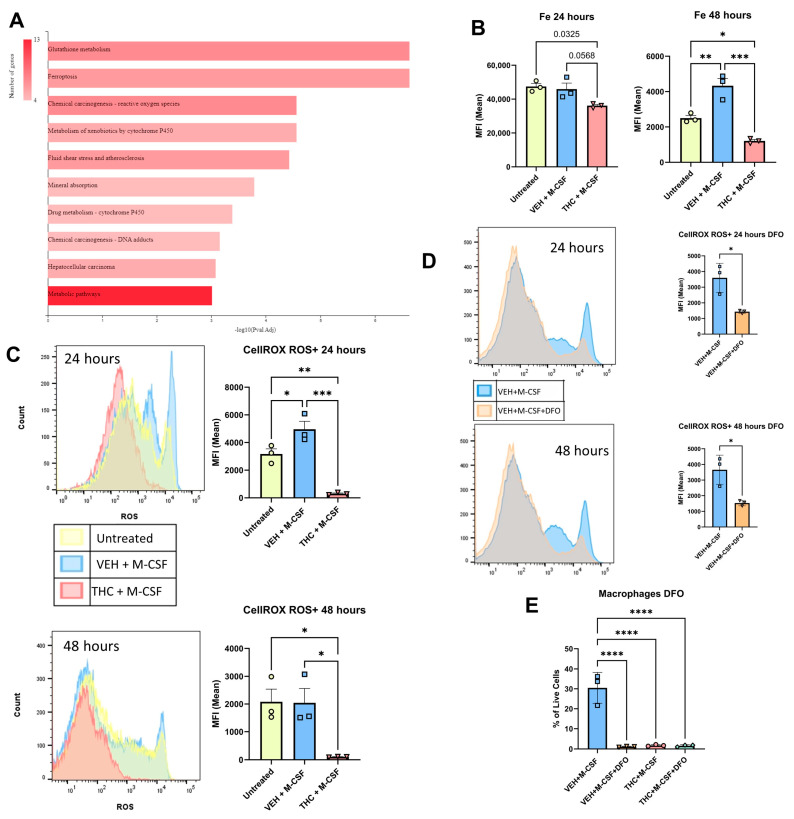
Role of iron and reactive oxygen species in the ability of THC to block macrophage differentiation. (**A**) KEGG pathway analysis of RNA-Seq using ShinyGO browser-based software v0.77. (**B**) Labile iron levels measured with PG SK at 24 and 48 h. (**C**) ROS levels and Live/Dead cells measured by CELLROX Deep Red and Sytox Blue at 24 and 48 h. (**D**) ROS levels and Live/Dead cells measured by CELLROX Deep Red and Sytox Blue at 24 and 48 h for VEH + M-CSF and VEH + M-CSF + DFO. (**E**) Graphical representation of the percentages of CD45 + CD11b + F4/80+ macrophages out of the total live cells as measured by Zombie UV in the presence of iron chelator DFO at 120 h. (**B**–**E**) n = 3, levels of statistical significance were assigned according to the following cutoffs: * *p* < 0.05, ** *p* < 0.01, *** *p* <0.001, and **** *p* < 0.0001.

Interestingly, the THC + M-CSF group displayed a unique expression profile involving antioxidant response (Figure 3L). Most of the genes upregulated following THC treatment have been reported to be increased following either NRF2 activation or downstream activation of Antioxidant Response Elements (AREs) [46]. Cannabinoids have been shown to induce antioxidant pathways, as well as having unique antioxidant molecular structure properties [47]. NRF2 is functionally unique in that it is normally expressed at constant levels and its association with KEAP1 leads to ubiquitination and proteasome-dependent degradation [48]. An alternative pathway that leads to the prevention of NRF2 degradation is the sequestering of KEAP1 by SQSTM1 [49]. *Sqstm1* was upregulated by THC (Figure 3F,L), as shown by Log2FC and rLOG normalization counts. THC and *Sqstm1* do have an established relationship, as it has previously been shown that THC has diminished effect in *Sqstm1* knockout mice [50]. THC also increased glutathione-related genes (Figure 3G,I,L) [51]. *Hmox1* and *Nqo1*, two central mediators of NRF2/ARE signaling [52], were also upregulated in the THC + M-CSF group (Figure 3H,L). The iron transporter *Slc40a1* and ferritin heavy chain gene *Fth1* were both increased in the THC + M-CSF group (Figure 3J,K). Finally, *Oas2* and *Irf7*, which had a higher expression in the VEH + M-CSF group, have previously been shown to be downregulated [53] when NRF2 is active in murine macrophages (Figure 3D,E). Overall, the mRNA signature of the VEH group matched that of an M-CSF-induced differentiating macrophage, while the THC group saw an upregulation in a significant number of ARE and iron-related genes. When the top 50 differentially expressed genes (filtered by counts and selected for Log2FC > 1.5 and *p*-value < 0.05) were run through the ShinyGO KEGG analysis software package v0.77 [54], the top two upregulated pathways were glutathione metabolism and ferroptosis (Figure 4A).

### 3.4. Reactive Oxygen Species and Fenton Reaction

To further investigate the potential role of ferroptosis, Fe^2+^ levels were measured using the PGSK assay. This fluorophore is quenched when binding iron; therefore, more iron results in lower fluorescence. At 24 h the THC + M-CSF group demonstrated insignificant changes in iron levels when compared to the untreated control group (*p* = 0.03) as well as the VEH + M-CSF group (*p* = 0.0568) (Figure 4B). At 48 h, however, the Veh + M-CSF group showed a significant decrease in iron levels in relation to the untreated BMDCS, while the THC + M-CSF group showed iron levels that were significantly higher than that seen in the VEH + M-CSF group as well as the untreated control group (Figure 4B). The regulation of iron levels is essential for homeostasis, and high levels can lead to the generation of damaging hydroxyl ions due to the Fenton reaction [55]. The Fenton reaction, which occurs between the switch of Fe^2+^ and Fe^3+^, can lead to a large increase in intracellular ROS, and commonly leads to ferroptosis [14]. THC has previously been shown to block the Fenton reaction in a controlled experiment at levels comparable to the commercial antioxidant BHT [56]. To assess ROS production within our model, we first used Sytox blue to select for live cells, then CELLRox Deep Red to assess ROS levels. A negative control of N-acetylcysteine and a positive control of Tert-butyl hydroperoxide are shown in Figure S2C. Surprisingly, THC significantly diminished ROS levels at both 24 and 48 h in the live cell group as measured by mean fluorescent intensity (MFI) (Figure 4C). Despite the THC-treated cells housing significantly high iron levels, THC was able to block the production of ROS.

ROS is essential for the differentiation of macrophages [10]. BHA, a synthetic antioxidant, has previously been shown to block M-CSF-induced macrophage differentiation by preventing the production of ROS [10]. Despite the increased iron observed in the THC group, ROS levels were significantly decreased by THC when compared to both the untreated and VEH + M-CSF groups, likely due to THC’s ability to block the Fenton reaction.

To further study the role of iron in macrophage differentiation in relation to ROS, the iron chelator, Deferoxamine (DFO), was added to the VEH + M-CSF and THC + M-CSF groups, and ROS levels and macrophage differentiation were examined. The addition of DFO did not result in any changes in the THC group (not shown), and thus, the VEH + M-CSF group was the focus of the next part of this study. At both 24 and 48 h, the addition of 50 uM DFO resulted in a significant decrease in ROS at both time points (Figure 4D and Figure S2B). At 120 h, 50 uM DFO blocked macrophage differentiation, with macrophage percentages similar to THC + M-CSF and THC + M-CSF + DFO (Figure 4E).

## 4. Discussion

Macrophages are an essential component of the immune response. THC’s effect on mature myeloid cells is well understood, but the effect on differentiating cells is not clearly defined. Our study demonstrated that when THC was added to BMDCs prior to the addition of M-CSF, macrophage differentiation was inhibited in a dose-dependent manner. THC is classically thought to operate through agonism at the CB1 and CB2 receptors. Interestingly, THC maintained its ability to block macrophage differentiation in CB1, CB2, and CB1/CB2 constitutive knockout mice. Recent studies have shown that THC is able to bind other GPCRs expressed on HSCs, including GPR18, GPR55, and A2aR. Treatment with antagonists specific to these receptors did not hinder THC’s ability to block macrophage differentiation. To confirm that a novel GPCR that THC could bind was not at play, PTX, which inhibits GPCR function, was also used, which did not alter THC’s ability to halt macrophage differentiation.

A time point of 48 h was selected for further RNA-seq analysis, as this was the start of the phenotypical shift from HSC to macrophage. This led to the discovery of a large increase in expression of NRF2-ARE-related genes in the THC group. Studies conducted over recent years have begun to establish a close association between the NRF2-ARE system and cannabinoids [47]. Some work suggests that this correlation is established by SQSTM1 (P62). SQSTM1 can bind and inhibit the action of NRF2 negative regulator E3 ubiquitin ligase KEAP1, thus preventing ubiquitin-directed degradation of NRF2 [49]. The close association of SQSTM1 and THC has been demonstrated in SQSTM1 knockout mice, in which THC demonstrated a diminished behavioral response [50].

The KEGG pathway analysis demonstrated that ferroptosis and glutathione metabolism pathways were upregulated in the THC group. The study of ferroptosis is a rapidly expanding field and the growing body of evidence suggests that it is implicated in multiple disease states. To investigate the potential role of ferroptosis in THC-mediated inhibition of macrophage differentiation, we utilized PGSK which measures intracellular iron and CellROX Deep Red which measures ROS. These assays are typically used to quantify histological samples but are ultimately limited by the number of cells per slide. Recent studies have shown that these assays can be used in single-cell suspensions and subsequently analyzed via flow cytometry, therefore allowing for the generation of high-throughput data [57,58]. THC treatment resulted in a significant increase in labile iron and nearly eliminated all ROS production at 24 and 48 h. These results suggested that THC’s ability to block ROS production may lead to the halting of macrophage differentiation, as ROS is essential to M-CSF-induced macrophage differentiation. A previous study by Zhang et al. showed that adding an antioxidant alongside M-CSF leads to blockage of macrophage differentiation and that ROS is necessary for proper M-CSF-mediated macrophage differentiation [10]. Additionally, this study showed that addition of H_2_O_2_ to the samples does not recover macrophage differentiation, potentially due to the increase in Catalase (CAT) levels, as seen in our THC group [10]. Research has shown that the extracellular addition of H_2_O_2_ is not effective in rescuing intracellular ROS as CAT begins the degradation process of H_2_O_2_ within seconds and the species is eliminated within minutes [59]. The significant increase in labile iron and marked lack of intracellular ROS post-THC treatment is a significant finding as increased intracellular iron normally leads to an increase in ROS due to the Fenton reaction [55]. To look further into the role of iron in the differentiation of macrophages, the use of the iron chelator revealed that iron is required for macrophage differentiation. Xie et al. have shown that the addition of iron can induce a macrophage-like phenotype in osteoclast precursor cells, as well as showing the effects DFO has on M-CSF and RANKL differentiation osteoclasts [60]. They found there was a switch between osteoclast and macrophage differentiation depending on the available iron levels. Our study has further shown that the chelation of iron can prevent the M-CSF-induced differentiation of macrophages.

In summary, this study has shown that THC, through both the induction of NRF2-ARE-related gene expression and its ability to block the Fenton reaction, attenuates ROS production, thus preventing M-CSF-induced macrophage differentiation. These results warrant further investigation into the role of SQSTM1 and NRF2-ARE-related genes in the immunomodulatory effects of THC, as well as further investigation into the role of iron in the differentiation of macrophages.

## Data Availability

Any raw data supporting the conclusions of this article will be made available by the authors.

## Supplementary Materials

The following supporting information can be downloaded at: https://www.mdpi.com/article/10.3390/antiox13080887/s1, Figure S1: Gating Strategy; Figure S2: Gating Strategy and ROS controls.
