# Supplementary material for: Delta-9-Tetrahydrocannabinol Blocks Bone Marrow-Derived Macrophage Differentiation through Elimination of Reactive Oxygen Species
Source: Antioxidants (Basel). 2024 Jul 23;13(8):887. doi: 10.3390/antiox13080887 (PMC11352128; doi:10.3390/antiox13080887)
Supplement: Supplementary file 1 [file antioxidants-13-00887-s001.zip › antioxidants-3069893-supplementary.pdf]

Figure S1. Representative flow cytometry pseudocolor plot and Preliminary flow gating strategy (A) Gating strategy for Figure 2A-D. Singlets>CD45+>CD45+CD11b+. (B) Gating strategy for Figure 2E-H. Singlets>Live>CD45+CD11b+. (C) Gating strategy for Figure 3A. Singlets>CD45+>CD45+CD11b+. (D) Gating strategy for Figure 3B. Singlets>CD45+>CD45+CD11b+.

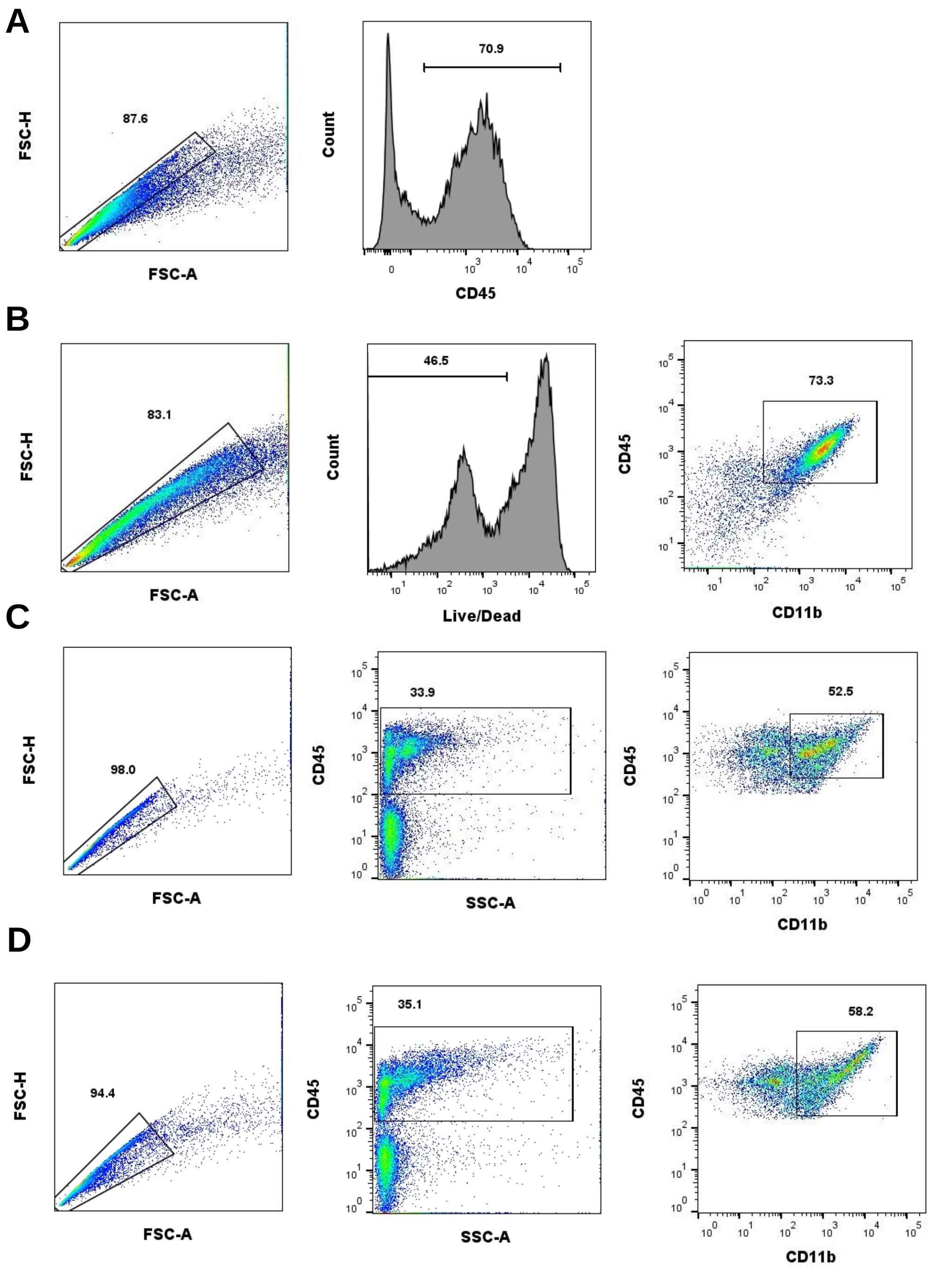
A
87.6
FSC-H
FSC-A
70.9
Count
CD45
B
83.1
FSC-H
FSC-A
46.5
Count
Live/Dead
73.3
CD45
CD11b
C
98.0
FSC-H
FSC-A
33.9
CD45
SSC-A
52.5
CD45
CD11b
D
94.4
FSC-H
FSC-A
35.1
CD45
SSC-A
58.2
CD45
CD11b

Figure S2. (A) Sytox blue gate for ROS analysis. (B) Representative flow plots for macrophage differentiation in the presence of DFO. (C) NAC and TBHP ROS controls of untreated bone marrow cells

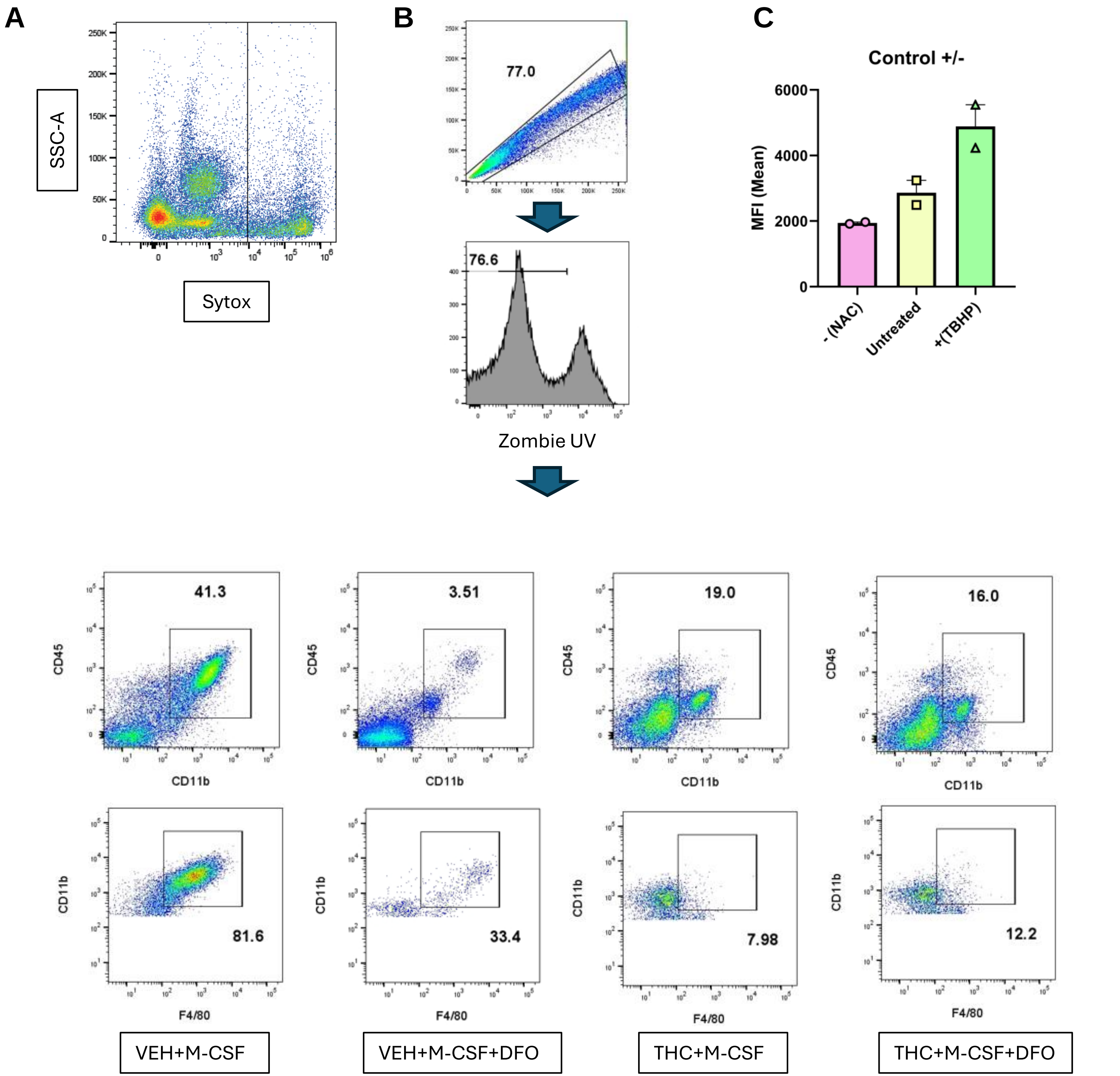
A
SSC-A
Sytox
B
77.0
76.6
Zombie UV
C
Control +/-
MFI (Mean)
- (NAC)
Untreated
+(TBHP)
41.3
3.51
19.0
16.0
CD45
CD11b
81.6
33.4
7.98
12.2
F4/80
VEH+M-CSF
VEH+M-CSF+DFO
THC+M-CSF
THC+M-CSF+DFO
